# Oncologic emergencies in a cancer center emergency department and in general emergency departments countywide and nationwide

**DOI:** 10.1371/journal.pone.0191658

**Published:** 2018-02-20

**Authors:** Zhi Yang, Runxiang Yang, Min Ji Kwak, Aiham Qdaisat, Junzhong Lin, Charles E. Begley, Cielito C. Reyes-Gibby, Sai-Ching Jim Yeung

**Affiliations:** 1 Department of Emergency Medicine, The University of Texas MD Anderson Cancer Center, Houston, Texas, United States of America; 2 Division of Management, Policy, and Community Health, The University of Texas Health Science Center at Houston School of Public Health, Houston, Texas, United States of America; 3 Department of Endocrine Neoplasia and Hormonal Disorders, The University of Texas MD Anderson Cancer Center, Houston, Texas, United States of America; Hospital de Santa Maria, PORTUGAL

## Abstract

**Background:**

Although cancer patients (CPs) are increasingly likely to visit emergency department (ED), no population-based study has compared the characteristics of CPs and non-cancer patients (NCPs) who visit the ED and examined factors associated with hospitalization via the ED. In this study, we (1) compared characteristics and diagnoses between CPs and NCPs who visited the ED in a cancer center or general hospital; (2) compared characteristics and diagnoses between CPs and NCPs who were hospitalized via the ED in a cancer center or general hospital; and (3) investigated important factors associated with such hospitalization.

**Methods and findings:**

We analyzed patient characteristic and diagnosis [based on International Classification of Diseases-9 (ICD-9) codes] data from the ED of a comprehensive cancer center (MDACC), 24 general EDs in Harris County, Texas (HCED), and the National Hospital Ambulatory Medical Care Survey (NHAMCS) from 1/1/2007–12/31/2009. Approximately 3.4 million ED visits were analyzed: 47,245, 3,248,973, and 104,566 visits for MDACC, HCED, and NHAMCS, respectively, of which 44,143 (93.4%), 44,583 (1.4%), and 632 (0.6%) were CP visits. CPs were older than NCPs and stayed longer in EDs. Lung, gastrointestinal (excluding colorectal), and genitourinary (excluding prostate) cancers were the three most common diagnoses related to ED visits at general EDs. CPs visiting MDACC were more likely than CPs visiting HCED to be privately insured. CPs were more likely than NCPs to be hospitalized. Pneumonia and influenza, fluid and electrolyte disorders, and fever were important predictive factors for CP hospitalization; coronary artery disease, cerebrovascular disease, and heart failure were important factors for NCP hospitalization.

**Conclusions:**

CPs consumed more ED resources than NCPs and had a higher hospitalization rate. Given the differences in characteristics and diagnoses between CPs and NCPs, ED physicians must pay special attention to CPs and be familiar with their unique set of oncologic emergencies.

## Introduction

Given the increasing incidence of and declining mortality rate for cancer worldwide, cancer patients (CPs) are increasingly likely to visit an emergency department (ED), either in cancer centers or general hospitals, at least once to obtain urgent care [1–3]. In previous studies, the ED-to-hospitalization rate of CPs (>50%) [1, 4] well exceeded that of non-CPs (NCPs) (11.9%) [5]. Moreover, as CPs have unique sequelae related to their disease and treatment, it is crucial for both general and cancer-specialist ED physicians to better understand the needs of CPs in emergent situations.

Research on CP ED visits has focused primarily on cancer type and chief complaints [1, 6, 7] end-of-life ED visits [3, 8, 9] or specific cancer types [10–12]. Most of this research has focused on commonalities among CPs; to our knowledge, none has compared the characteristics of CPs and NCPs who visit the ED, either in cancer centers or general hospitals. Moreover, although several studies have shown that hospitalization via the ED is a clinically important marker of poorer prognosis for CPs [13–15], no population-based study has examined factors associated with CP hospitalization via the ED.

In this study, we (1) compared characteristics and diagnoses between CPs and NCPs who visited the ED in a cancer center or general hospital; (2) compared characteristics and diagnoses between CPs and NCPs who were hospitalized via the ED in a cancer center or general hospital; and (3) investigated important factors associated with such hospitalization.

## Methods

### Data collection

We collected data on the characteristics of visitors to the ED at The University of Texas MD Anderson Cancer Center in Houston, Texas, visitors to EDs at general hospitals in Harris County, Texas (which includes Houston), and ED visitors assessed in the US National Hospital Ambulatory Medical Care Survey (NHAMCS). Our study was conducted under a clinical research protocol (DR08-0066) approved by the MD Anderson Institutional Review Board and in compliance with Health Insurance Portability and Accountability Act regulations. As this was a retrospective data review, informed consent requirements were waived.

MD Anderson is a specialized referral center for cancer care. Its ED handles ~22,000 patient visits per year; >90% of the ED visitors are MD Anderson patients. Study data (hereafter, “MDACC”) were obtained from the institution’s tumor registry and electronic medical records.

Countywide data were collected from 24 general hospital EDs located in Harris County (hereafter, “HCED”). Harris County had an estimated 4.25 million residents in 2012 [16]. A partnership among the Harris County Hospital District, The University of Texas School of Public Health, and Gateway to Care, established to monitor ED use in the Houston 911 service area [17], provided data from approximately two thirds of the hospital-based ERs within this region. This database contains up to ten International Classification of Diseases, 9th Revision, Clinical Modification (ICD-9) codes per visit.

NHAMCS includes a retrospective national probability sample survey of visits to hospital outpatient clinics and EDs in 50 states and the District of Columbia [18]. The Emergency Department Summary uses a manually extracted sample to estimate national ED data.

All three databases had basic demographic and clinical information for every ED visit patient, including age, sex, race, cancer type, disposition (admitted, discharged, died, or other), dates and times related to ED visit, insurance (private, government-paid, other/unknown), and method of arrival at ED (ambulance, clinic visit, walk). Residence ZIP code was available in the MDACC and HCED databases.

### Statistical analysis

All statistical analyses were performed using R software (version 3.2.2, The R Foundation, http://www.r-project.org).

Data from the time period between January 1, 2007 and December 31, 2009 were analyzed. We used two different methods to define CPs: for MDACC, we examined the institutional tumor registry to determine whether a patient had cancer and, if so, what kind of cancer they had. For HCED and NHAMCS, CPs were determined by association with ICD-9 codes for malignancy, as described by Mayer et al [6]. This method was also applied to the MDACC data, to compare the performance of these two methods and identify potential limitations.

All ICD-9 codes for ED visitors were divided among the standard 19 ICD-9 categories, and the percentage frequencies of these code categories were summarized for ED visits and admissions through EDs.

The Charlson Comorbidity Index (CCI) is a scoring system that is widely used to evaluate the comorbid conditions for prognostic purposes [19]. We calculated the CCI using available ICD-9 codes and the “icd9Charlson” function of the R package “icd9” (version 1.3.1).

Random forests is an ensemble learning method for classification, regression, and other tasks that constructs multiple decision trees at training time and outputs the class that is the mode of the classes (classification) or mean prediction (regression) of individual trees. Types of cancer, ICD-9 code categories, important symptoms, and unusual but emergent symptoms were chosen as factors for random-forest analysis to evaluate their importance in the hospitalization decision, controlled for demographics (eg, age, sex, race) and ED visit characteristics (eg, arrival by ambulance, visit during business days/business hours, length of stay). Two random-forest implementations in R were used: “randomForest” (version 4.6–12) and “h2o.randomForest” (h2o version 3.10.0.8). Internal validation of the prediction by each random-forest model was performed by randomly dividing each data set into 75% for training and 25% for validation; the performance of each model was assessed by a receiver-operating characteristic (ROC) curve and its area under the curve (AUC). Ranked lists of relative importance of top contributing factors from randomForest and h2o.randomForest were then combined by rank aggregation (R package “RankAggreg”, version 0.5) to assess the association of those factors with hospitalization through the ED.

## Results

### Patient characteristics

We identified ~3.4 million ED visits between 2007 and 2009. In the MDACC database, there were 47,245 ED visits, including 44,143 visits by CPs [93.4%] and 3,102 visits by NCPs per the tumor registry, or 32,477 visits by CPs and 14,768 visits by NCPs per ICD-9. In the HCED database, there were 3,248,973 ED visits (44,583 CPs [1.4%] and 3,204,390 NCPs); in the NHAMCS database, there were 104,566 ED visits (632 CPs [0.6%] and 103,934 NCPs).

**Fig 1:**
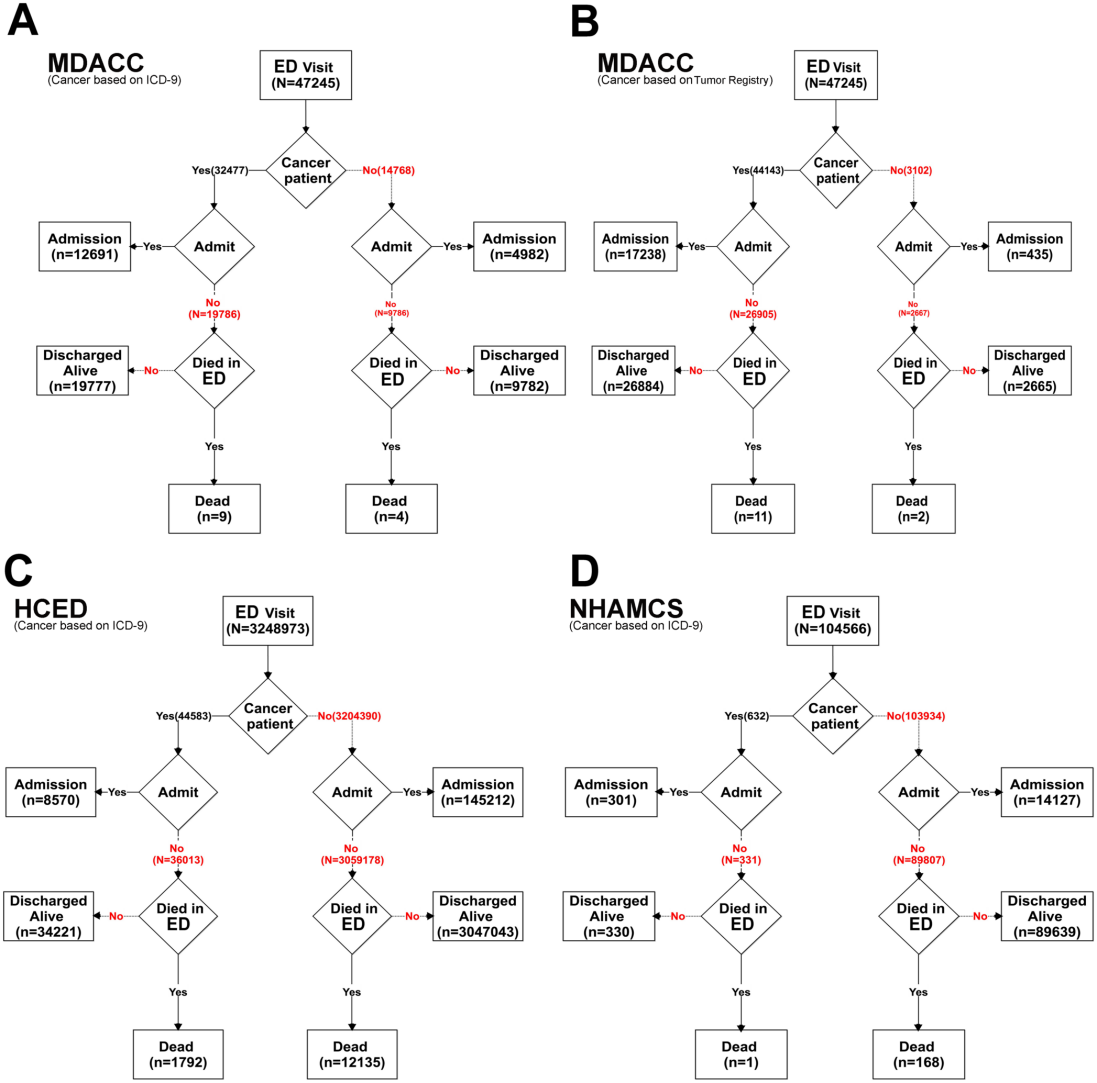
Numbers of visitors who were discharged, hospitalized, or died in ED between 2007 and 2009. (A, B) MDACC. (C) HCED. (D) *NHAMCS*. CPs were discovered by association with ICD-9 codes for malignancies (A, C, D) or by a tumor registry (B).

In the MDACC database, 17,673 ED visitors were hospitalized (17,238 CPs, 435 NCPs per tumor registry, or 12,691 CPs, 4,982 NCPs per ICD-9); in the HCED database, 153,782 were hospitalized (8,570 CPs, 145,212 NCPs); and in the NHAMCS database, 14,428 were hospitalized (301 CPs, 14,127 NCPs) (Fig 1). CPs as defined by the tumor registry accounted for nearly 95% of patients visiting the MD Anderson ED, but CPs comprised only 1% of patients visiting the general EDs. A higher hospitalization rate was found for CPs than for NCPs in each database (MDACC: 39.1% vs 14.0% per tumor registry; HCED: 19.2% vs 4.5%; NHAMCS: 47.6% vs 13.6%; *P*<0.01).

**Fig 2:**
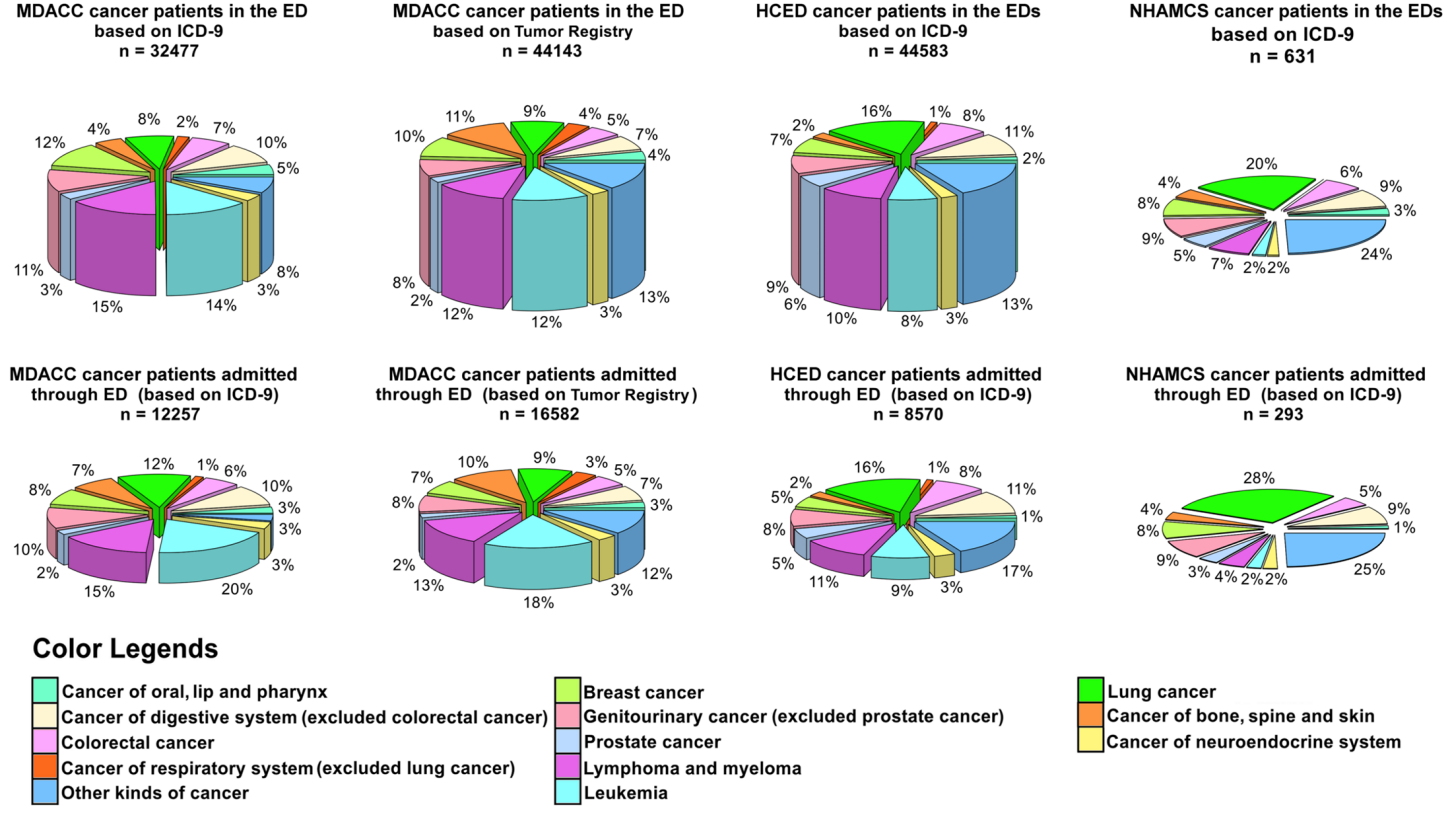
Percentages of various cancer types in ED visitors and those who got admission. Percentages of various cancer types in patients who visited the ED (top row) and in those who were admitted through the ED (bottom row). The thickness of each pie is scaled to represent the total number of CPs in each data set.

Fewer CPs with hematological malignancies (leukemia, lymphoma/myeloma) visited general EDs than visited the MD Anderson ED (Fig 2). Lung, gastrointestinal (excluding colorectal), and genitourinary (excluding prostate) cancers were the three most common cancer diagnoses related to ED visits at general EDs, apart from the miscellaneous category “other cancers” (several rare cancers and metastatic cancer with an unknown primary tumor). Among hospitalized CPs, leukemia, lymphoma/myeloma, and lung cancer were the three most common cancer diagnoses related to ED visits in MDACC, whereas lung and gastrointestinal (excluding colorectal) cancer were the most common cancer diagnoses related to ED visits in HCED and NHAMCS. For all cancer types, CP admission rates in HCED were the lowest among the three data sets (S1 Fig). The admission rates in NHAMCS were higher than those in MDACC for all cancer types except colorectal cancer, leukemia, cancer of the lip/oral cavity/pharynx, lymphoma/myeloma, and cancer of the respiratory system not including lung cancer.

### Age

In the pooled data from all three data sets, CPs visiting the ED were older than NCPs (CPs: 57.87±18.47 years; NCPs: 33.16±24.12 years; *P*<0.01); the same was true for admitted patients (CPs: 58.61±17.73 years; NCPs: 50.84±26.18 years; *P*<0.01). After defining seven age groups, one for every 15 years of life, differences between CPs and NCPs in the percentage distributions of ED visits and admissions through EDs became apparent in all three databases (S2 Fig). NCP children and young adults were the most common ED visitors in HCED and NHAMCS. Because most NCP ED visitors in the MDACC database were employees, visitors, and family and friends of CPs being treated at MD Anderson, the percentage distribution was very low for the pediatric group and peaked at 46–60-years of age. Among CPs and NCPs admitted through the ED, most were 46–75 years of age.

**Fig 3:**
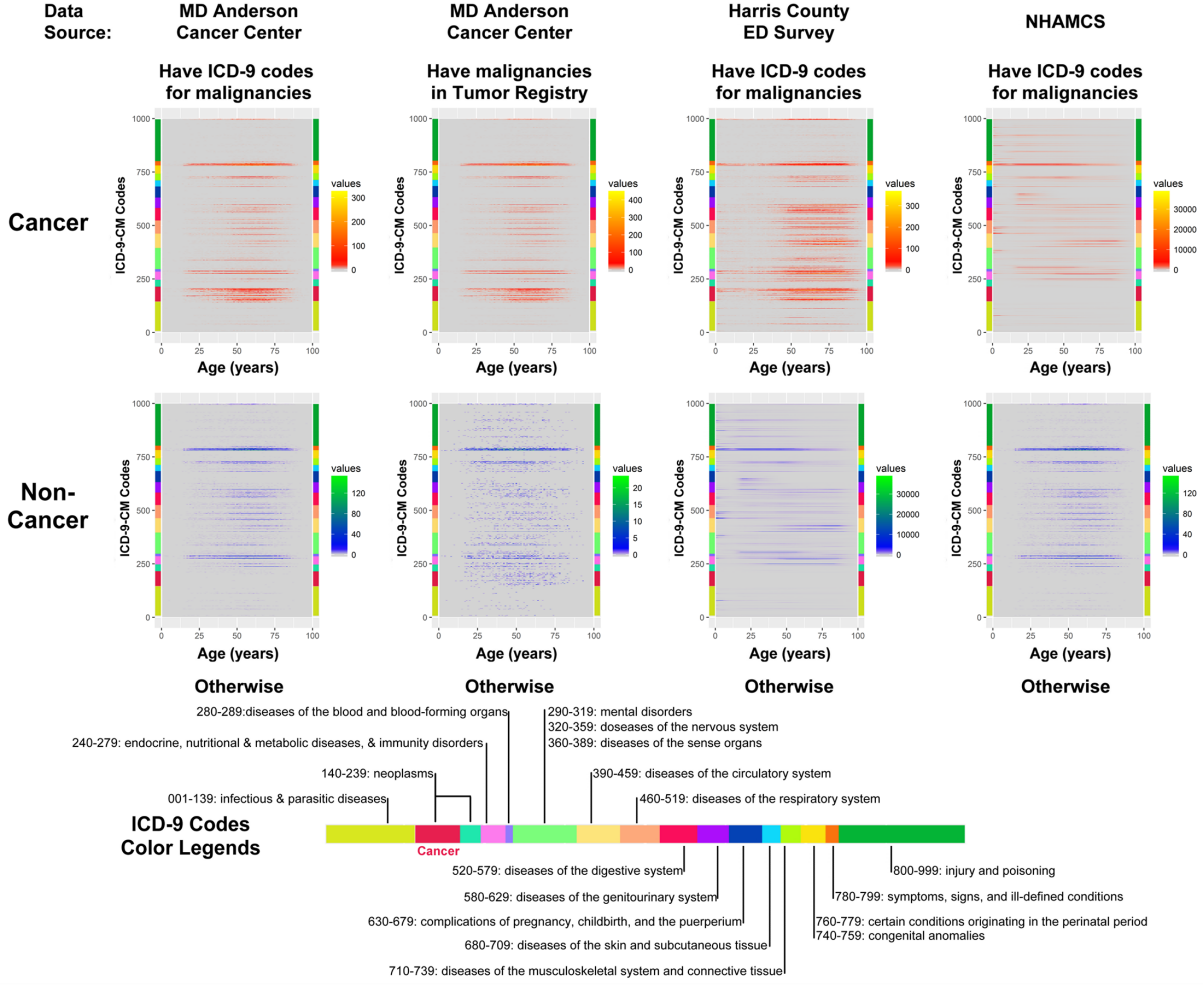
Heat maps of ICD-9 codes at different ages for CPs and NCPs. The color key shown to the right of each panel relates color intensity to the number of patients.

CPs and NCPs visiting the ED had various ICD-9 diagnoses across age ranges (Fig 3). For example, apart from visiting the ED for symptoms and cancer diagnoses, CPs aged 50–75 years visited the ED for endocrine/metabolic, circulatory, respiratory, and gastrointestinal disease. However, NCPs aged 50–75 years visited the ED mainly for endocrine/metabolic and circulatory disease, in addition to symptoms.

### Residence and insurance

**Fig 4:**
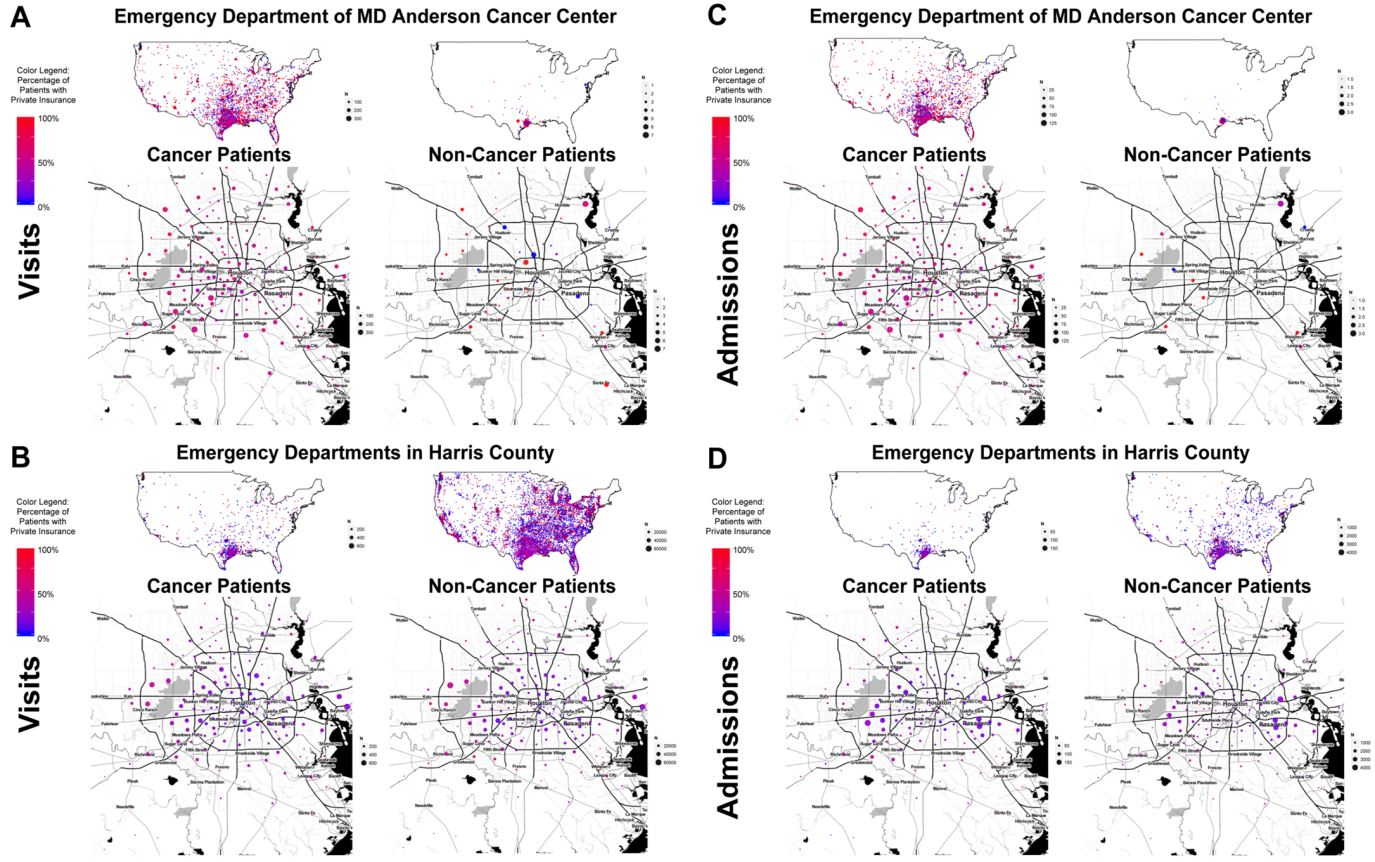
Relationship of insurance and geographic location of residence of ED visitors and those got admission. Relationship of insurance and geographic location of residence of CPs and NCPs visiting the ED and admitted through the ED (single institution and countywide data only). In each panel, the dots mark the location of the residence ZIP code on maps of the United States (above) and maps of Harris County (below). The size of the dot represents the number of patients (see size key at right of map). The color of the dot represents the percentage of patients with private insurance (see color key at left of map). (A) Data for MDACC CPs and NCPs who visited the ED. (B) Data for HCED CPs and NCPs who visited the ED. (C) Data for MDACC CPs and NCPs who were hospitalized through the ED. (D) Data for HCED CPs and NCPs who were hospitalized through the ED.

MD Anderson is a comprehensive cancer center with a national referral base; several major hospitals in the Texas Medical Center are also major tertiary referral centers for a variety of nonmalignant diseases. As residence ZIP codes were available in the MDACC and HCED databases, we used that data to visualize and compare the relationships between geographic location of residence and insurance type for the patients who visited EDs and those who were admitted (Fig 4).

Most of the MDACC ED visitors were CPs from various parts of the United States and had private insurance, whereas the MDACC NCPs were mainly from Harris County and its vicinity and were covered by government insurance (Fig 4A). In contrast, most of the HCED ED visitors were NCPs from various parts of the United States, while the HCED CPs were mainly from Harris County and its vicinity. The percentages of private insurance and government insurance were equal in HCED ED visitors overall (both CPs and NCPs) (Fig 4B). For both the MDACC CPs and NCPs admitted to the hospital, the patterns were similar to those seen in ED visitors (Fig 4C). For the HCED admitted patients, most admitted patients were from Harris County and its vicinity and were covered by government insurance (Fig 4D).

### Time

All the ED visits and admissions via ED in all three databases were examined for variations by time of the day, day of the week, day of the month, and month of the year. The time of the day for ED visits and admissions ranged from the fewest visits and admissions in the early morning hours (4 am to 7 am) to peak numbers in midafternoon (12 pm to 3 pm) for both CPs and NCPs (S3 Fig, upper panels). As for the day of the week (S3 Fig, lower panels), a decrease in visits and admissions from Monday to Sunday was observed for CPs (with the lowest numbers on Saturday); however, no similar trend was seen for NCPs. Moreover, compared with NCPs, CPs had longer ED stays (CPs: 11.55±10.22 hours; NCPs: 6.03±7.93 hours: *P*<0.001] in all three databases, indicating that the severity of illness in CPs was greater than that in NCPs and that more medical resources were consumed by CPs.

### Factors associated with admission through EDs in CPs and NCPs

Among patients admitted through EDs, CPs generally had higher admission rates than NCPs across the large majority of diagnostic groups (S4 Fig). The admission rates of CPs in MDACC agree with those in NHAMCS for most diagnostic groups.

Random forest methodology was used to identify important factors associated with the decision to admit for all ED visitors. After optimizing the numbers of trees in the random forest analysis, we ran the “randomForest” R package to determine appropriate cutpoints for age, length of stay in ED, and comorbidities (CCI) in CPs and NCPs. As already shown in S2 Fig, the influence of age on admission was different between CPs and NCPs. Since the admission rate might increase with increasing length of stay in the ED and CCI in ED visitors, we chose a length of stay >5 hours and a CCI >2 as cutpoints for conversion into categorical covariates for further analysis.

**Fig 5:**
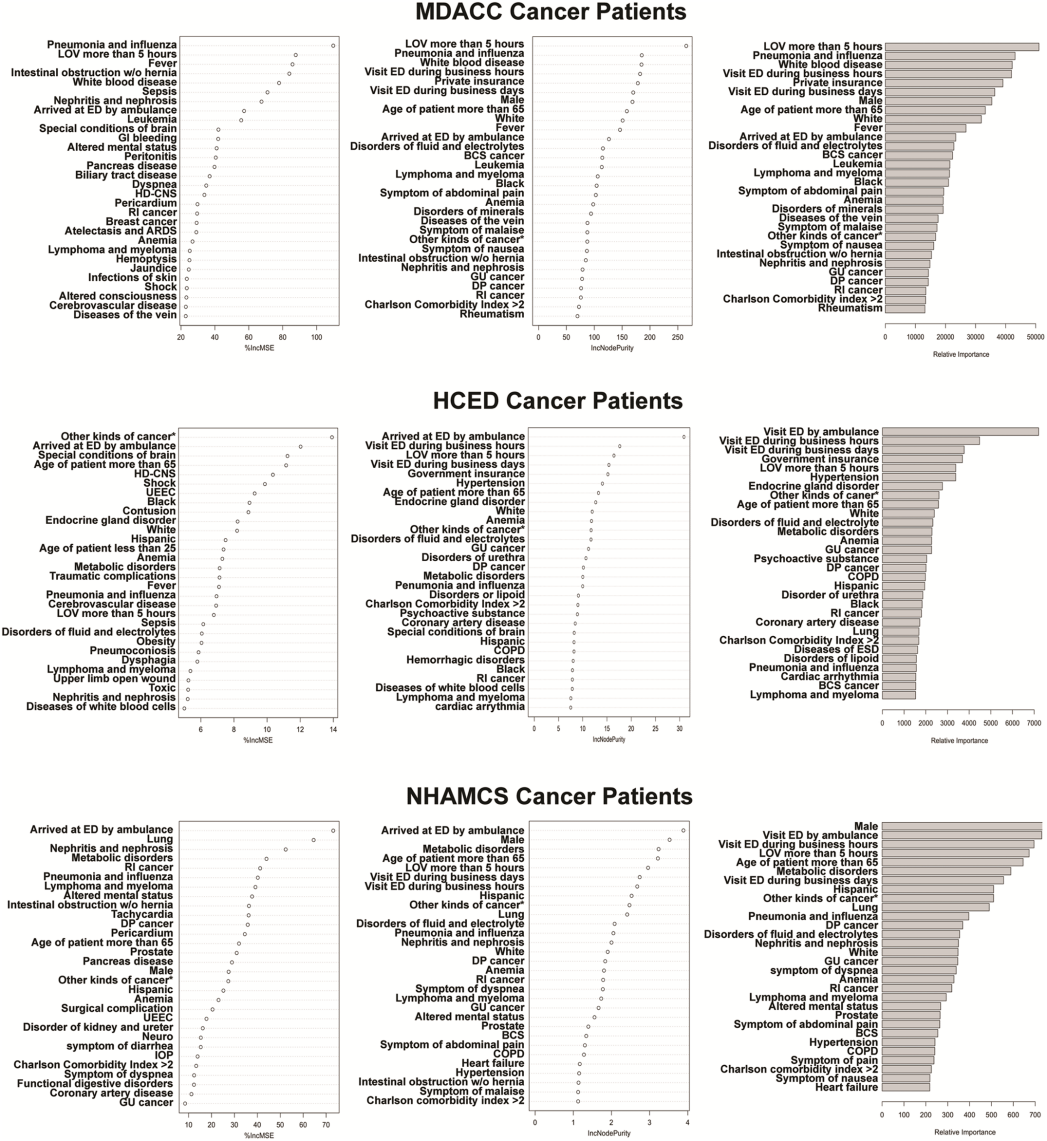
Factors associated with CP admission through the ED. For each database,the top 30 factors associated with the ED admission were ranked by the average percentage increase in mean squared error (%IncMSE) (left panels) or the increase in node purity (IncNodePurity) as calculated by the residual sum of squares (middle panels) using the R package “randomForest”. The relative importance results of factors were identified by random forest using the R package “h2o” (right panels).

**Fig 6:**
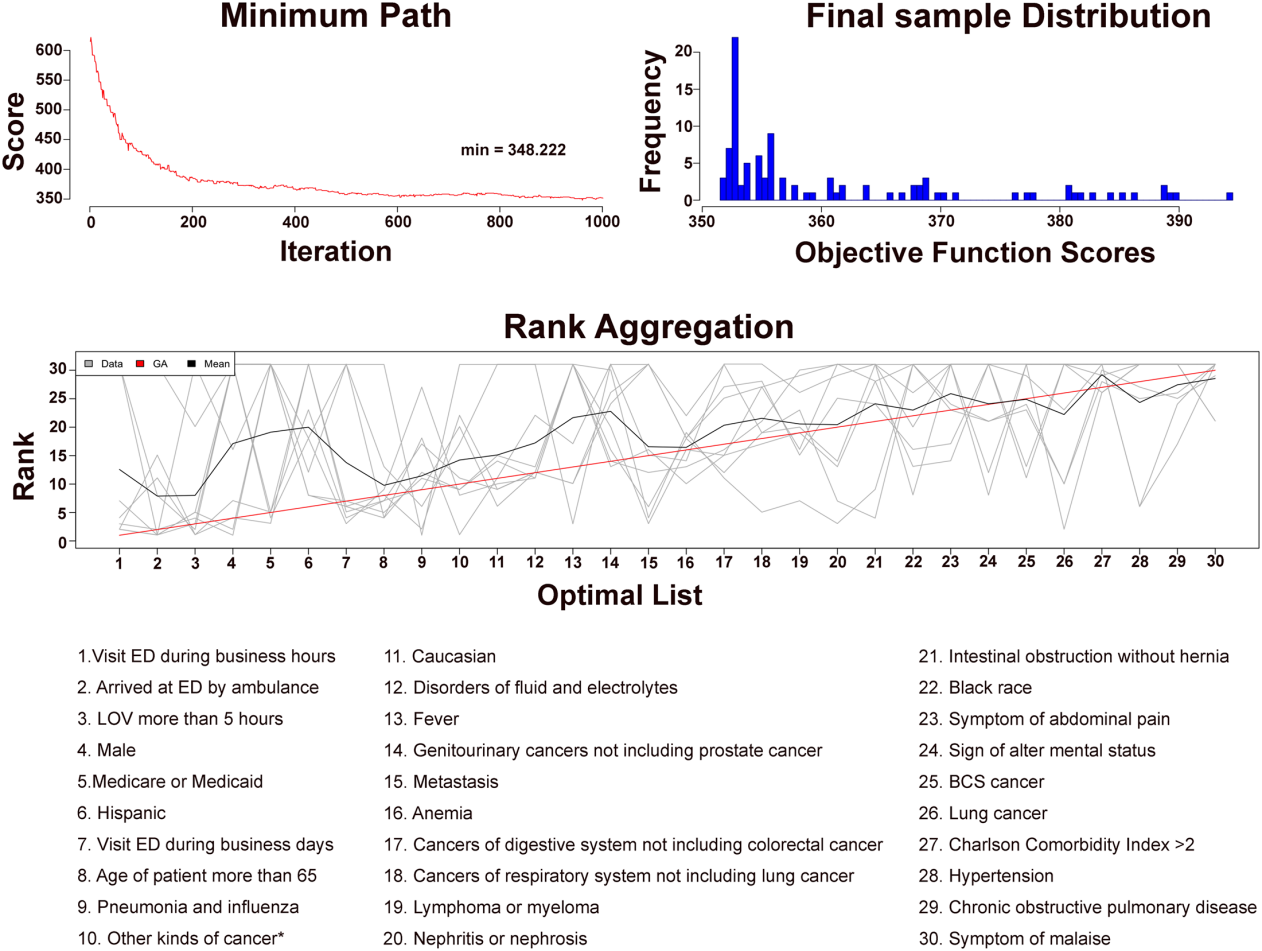
Rank aggregation of the top 30 factors associated with admission through the ED for CPs. The ranked lists from Fig 5 were aggregated into one list. Result of Rank Aggregation was shown with a genetic algorithm (GA) score of 348.2.

To avoid bias in favor of continuous variables and variables with multiple categories, nonbinary variables were converted to binary categorical variables as described above and entered into classification prediction by random forest. ROC analysis of internal validation results showed excellent prediction of admission by the random forest models (all AUCs >0.80). The random forest analysis identified and ranked the relative importance of factors that predict the admission of CPs through the ED in all three databases, each generating three lists: one by h2o.randomForest, one based on the average percentage increase in mean squared error by randomForest, and one based on mean decrease in node impurity by randomForest (Fig 5). Similarly, nine ranked lists of factors that predict admission through the ED were obtained for NCPs (S5 Fig). For CPs, the ordered ranks from the nine lists in Fig 5 were aggregated into one list using the R package “RankAggreg” (version 0.5) (Fig 6). Because NCPs at MDACC were low in number and very different from NCPs in general EDs, a final aggregated ranked list based on the lists from HCED and NHAMCS in S5 Fig was generated for NCPs (S6 Fig). Besides patient and care delivery characteristics (such as age, sex, race, arrival by ambulance, visit during business days/business hours, and length of stay), the top three predictors of admission through ED for CPs were pneumonia and influenza, disorders of fluid and electrolytes, and fever, in contrast to coronary artery disease, cerebrovascular disease, and heart failure for NCPs.

## Discussion

Our study was the first of its kind to include data for both CPs and NCPs from databases at multiple geographic levels (single institution, regional, and national) in consecutive years (2007–2009). The large dataset (3,400,152 visits) allowed in-depth analysis of the characteristics of patients who visited EDs and who were hospitalized via EDs in detail not achievable before.

Previous studies have indicated that lung, colorectal, and breast cancers were the most common in general EDs [1, 6], whereas hematological, breast, and gastrointestinal cancers were the most common in comprehensive cancer center EDs [13], similar to our results. One of our interesting findings is that although the number of ED visits by CPs with neuroendocrine cancer was relatively small, they had a very high admission rate. Perhaps emergency physicians should pay special attention to CPs with neuroendocrine cancer.

Mayer et al [6] found that about 44.9% of ED visits happened during clinic hours, consistent with our results. The number of CPs who visited the ED and were subsequently hospitalized decreased from Monday to Sunday in our study. Although geographic variations in the patterns of ED visits and admission times may exist, our results provide regional and national trends that may help health care administrators to interpret local trends and allocate ED resources appropriately.

We analyzed a large number of factors for their association with hospitalization through the ED. We found that pneumonia and influenza, disorders of fluid and electrolytes, and fever were important predictive factors for hospitalization, in addition to common factors such as age, sex, race, arrival by ambulance, visit during business day/business hours, and length of stay. Although pain was the most common complaint in CPs in previous ED studies [3, 6, 14, 15] and the most common symptom in our study, most visitors were released from the ED without hospitalization. This suggests that many CPs may be able to avoid an ED visit if they can get effective pain management in outpatient clinics. Fever and respiratory problems were common in CPs with lung infection; they were important factors associated with hospitalization in CPs, especially those with hematological malignancies. Disorders of fluid and electrolytes were important factors associated with hospitalization.

Our study had several limitations. First, because certain patient information was missing from the databases, we could not determine whether any patients paid multiple visits to the same or different EDs or identify CPs who may have visited both MD Anderson and general EDs. Second, our comparison of CP identification via ICD-9 versus the tumor registry showed that ICD-9 missed some CPs; nonetheless, the number of CPs misclassified as NCPs based on ICD-9 codes was small and did not change the overall profile of NCPs in general EDs. Despite these limitations, the analyses produced reliable results generally representative of CPs and NCPs, due to the large sample.

This study provided a three-tiered analysis of CPs and NCPs who visited the ED and who were hospitalized via the ED in a comprehensive cancer center, countywide, and nationwide. For ED visitors, pneumonia and influenza, disorders of fluid and electrolytes, and fever were important predictors for CP hospitalization, whereas coronary artery disease, cerebrovascular disease, and heart failure were important factors for NCP hospitalization. Given that CPs were older and remained in the ED longer attests to the high level of complexity in the emergency care of CPs compared with NCPs. CPs visiting the MD Anderson ED, including those who were hospitalized, were more likely to be privately insured than any of the other patients in our sample, suggesting an influence of patients’ choice of health care providers when adequate financial resources were available.

ED physicians must pay special attention to CPs and be familiar with the unique set of challenges imposed by oncologic emergencies. The need to promote and facilitate future research on oncologic emergencies was recognized by the National Cancer Institute, which sponsored the formation of the Comprehensive Oncologic Emergencies Research Network (CONCERN) [20]. Prospective data collection about the epidemiology of oncologic emergencies is in progress.

## Conclusions

CPs consumed more ED resources than NCPs and had a higher hospitalization rate. Given the differences in characteristics and diagnoses between CPs and NCPs, ED physicians must pay special attention to CPs and be familiar with their unique set of oncologic emergencies.

## Supporting information

S1 Fig Rates of hospital admission through the ED for CPs, by type of cancer. (TIF)

S2 Fig Patient age distribution for ED visits and for admissions through the ED. Patient age distribution for ED visits and for admissions through the ED. Patients were divided into seven age ranges (x-axis).
(TIF)

S3 Fig Time of day and day of week for ED visits and admissions through the ED. Patients were divided into six 4-hour periods by the time of the day they arrived in the ED (upper panels) and by the day of the week they arrived in the ED (lower panels).
(TIF)

S4 Fig Admission rates through the ED for CPs and NCPs, by ICD-9 diagnostic category. (TIF)

S5 Fig Factors associated with NCP admission through the ED. For each database, the top 30 factors associated with the ED admission were ranked by the average percentage increase in mean squared error (%IncMSE) (left panels) or the increase in node purity (IncNodePurity) as calculated by the residual sum of squares (middle panels) using the R package “randomForest”. The relative importance results of factors were identified by random forest using the R package “h2o” (right panels).
(TIF)

S6 Fig Rank aggregation of the top 30 factors associated with admission through the ED for NCPs. Rank aggregation of the top 30 factors associated with admission through the ED for NCPs who visited the ED (HCED and NHAMCS only). The ranked lists from S5 Fig were aggregated into one list. Result of Rank Aggregation was shown with a genetic algorithm (GA) score of 351.3.
(TIF)

## Abbreviations

AUC: area under the curve
BCS: bone/connective tissue/skin
CCI: Charlson Comorbidity Index
CP: cancer patient
ED: emergency department
HCED: Harris County database
ICD-9 and ICD-9-CM: International Classification of Diseases, 9th Revision, Clinical Modification
LOV: length of visit
MDACC: The University of Texas MD Anderson Cancer Center database
NCP: non-cancer patient
NHAMCS: National Hospital Ambulatory Medical Care Survey database
ROC: receiver-operating characteristic
