# Supplementary figures and images for: Oncologic emergencies in a cancer center emergency department and in general emergency departments countywide and nationwide
Source: PLoS One. 2018 Feb 20;13(2):e0191658. doi: 10.1371/journal.pone.0191658 (PMC5819770; doi:10.1371/journal.pone.0191658)

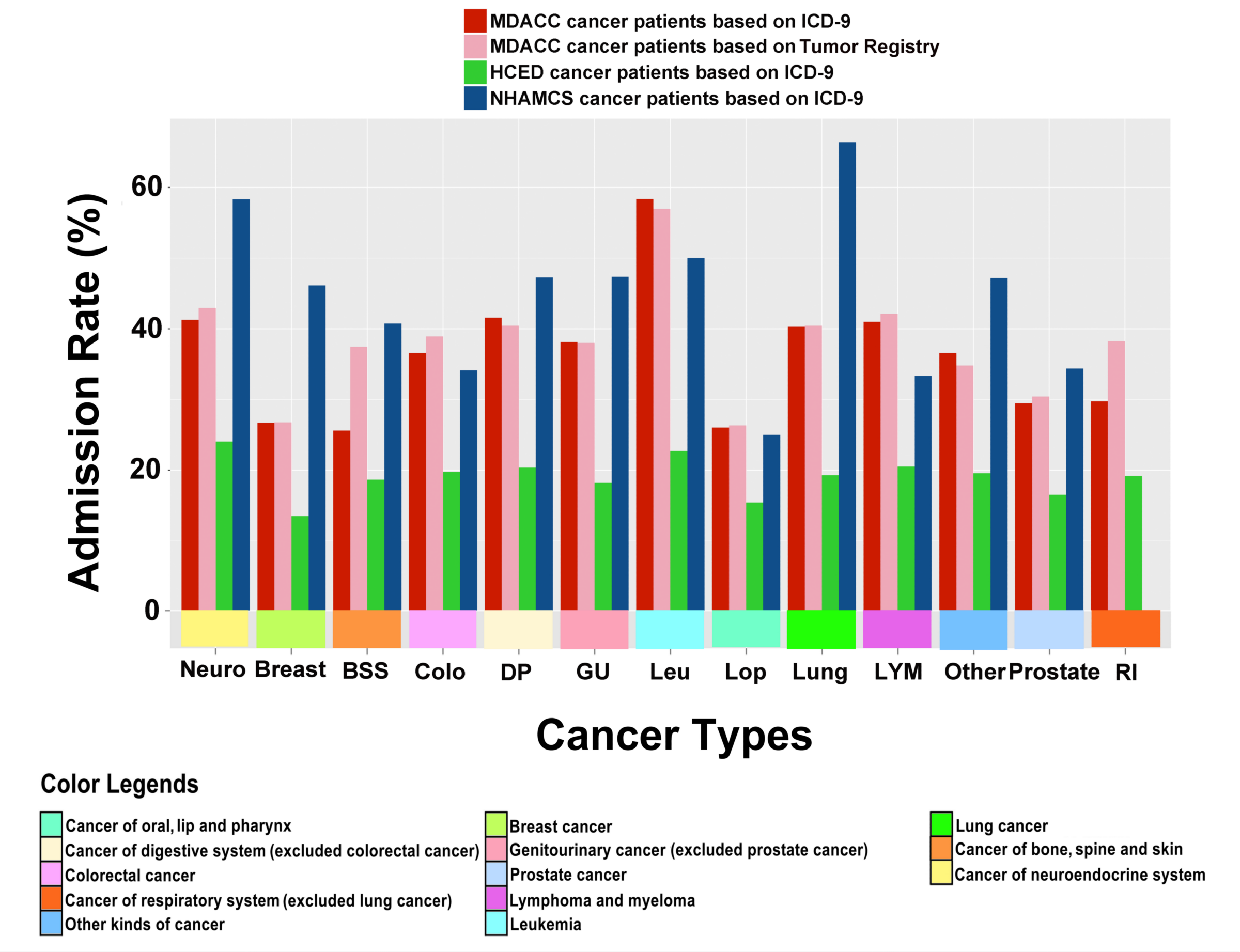

Supplement: S1 Fig — (TIF) [file pone.0191658.s001.tif]

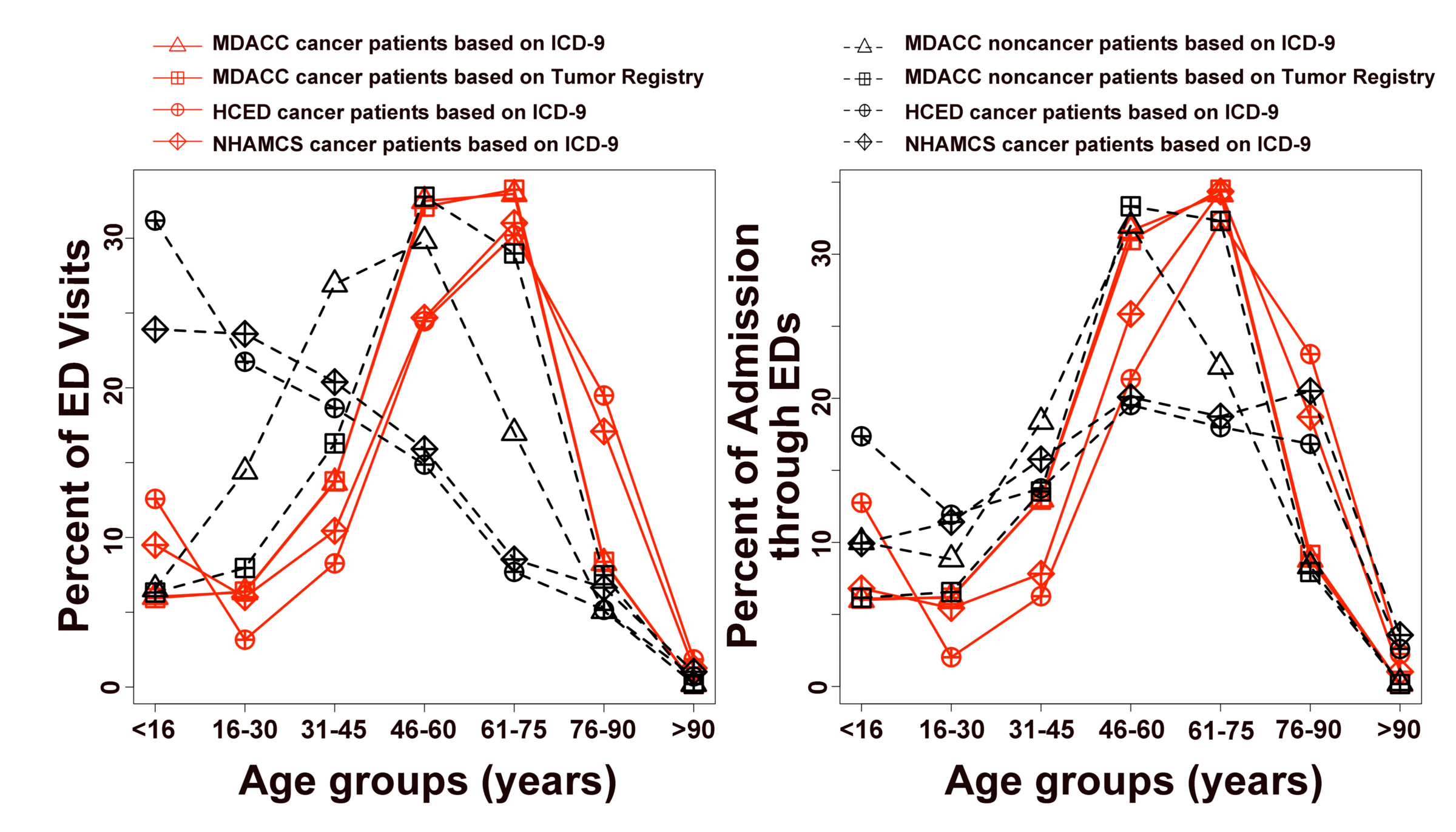

Supplement: S2 Fig — Patient age distribution for ED visits and for admissions through the ED. Patients were divided into seven age ranges (x-axis). (TIF) [file pone.0191658.s002.tif]

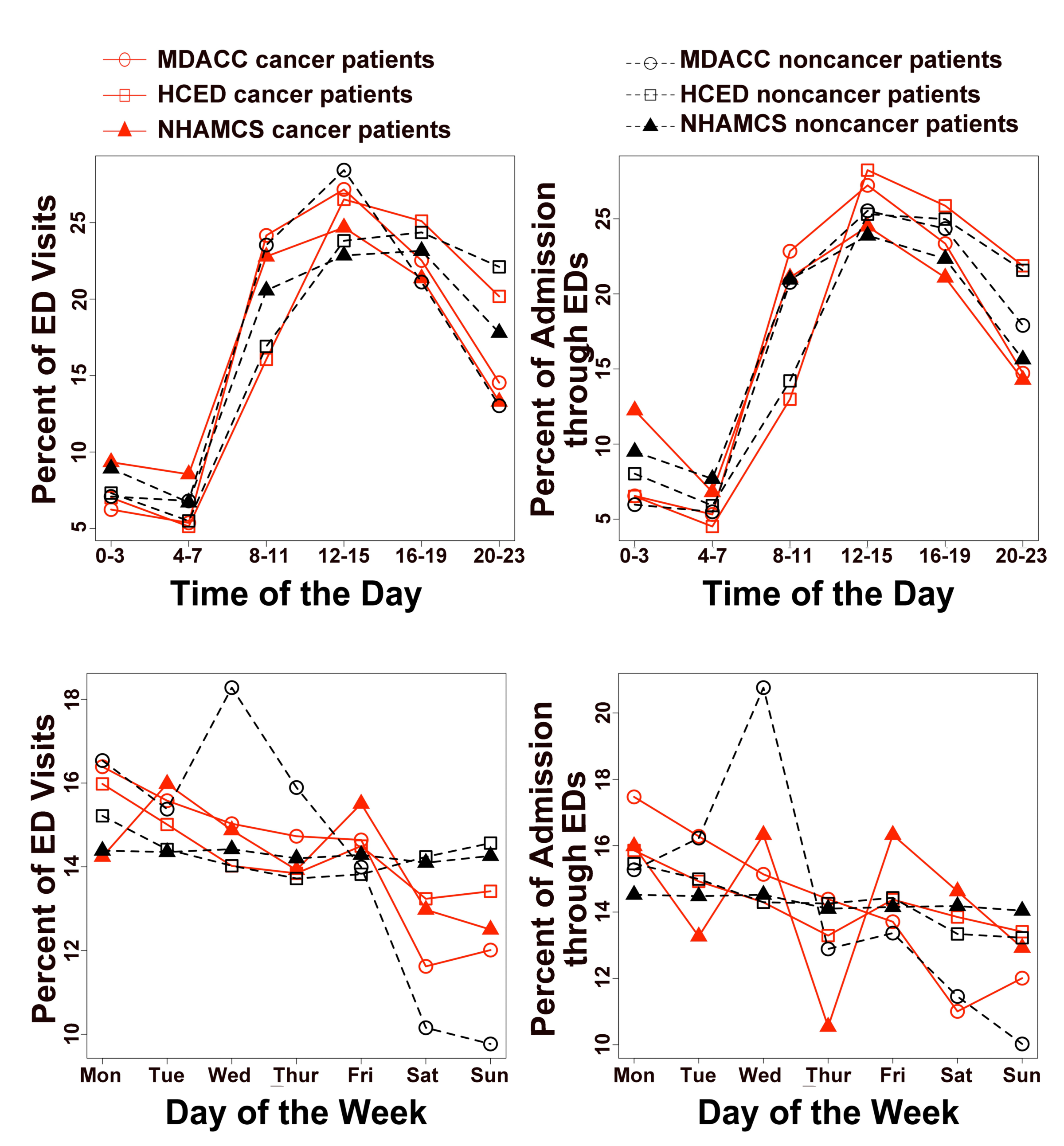

Supplement: S3 Fig — Patients were divided into six 4-hour periods by the time of the day they arrived in the ED (upper panels) and by the day of the week they arrived in the ED (lower panels). (TIF) [file pone.0191658.s003.tif]

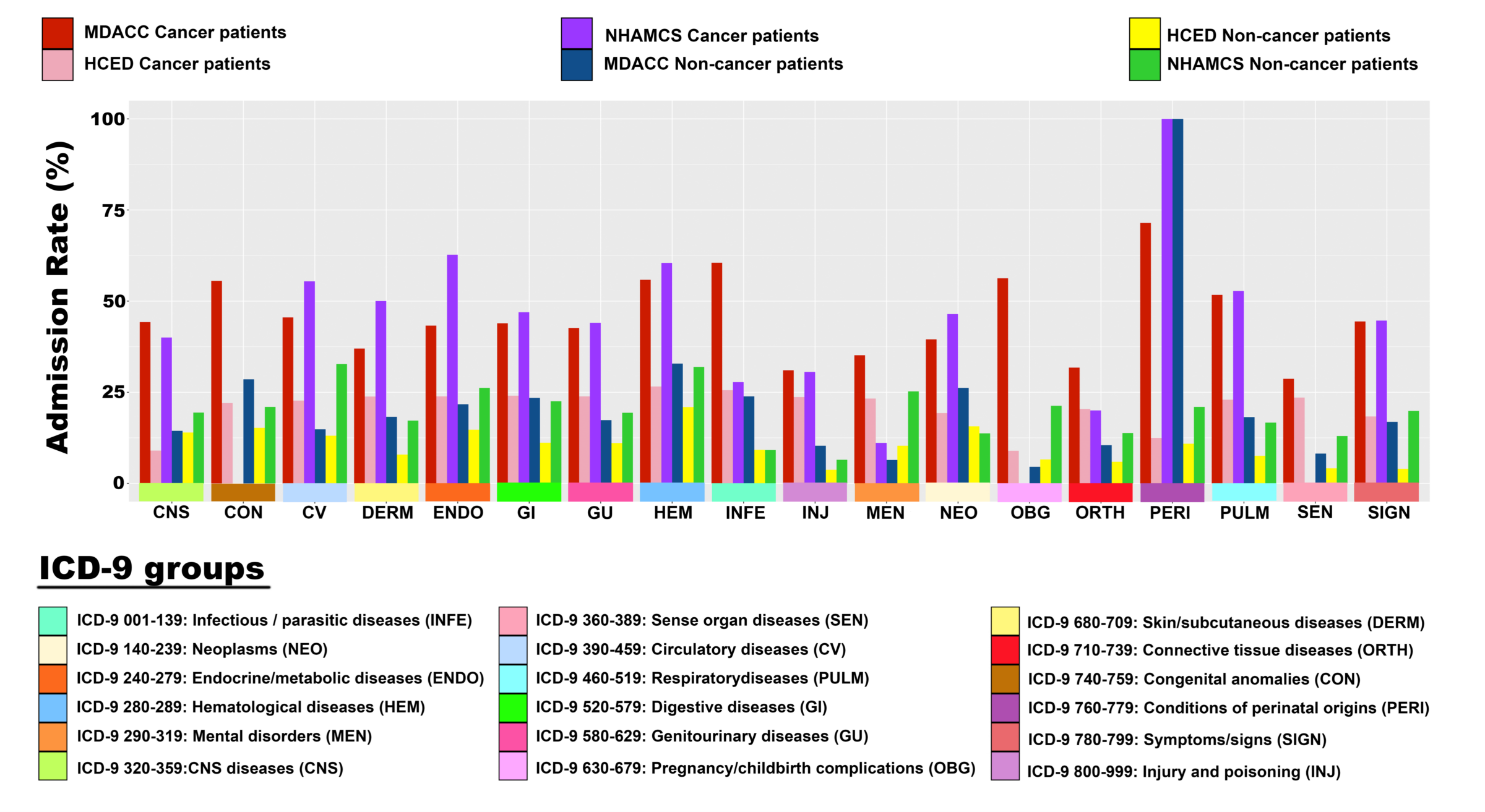

Supplement: S4 Fig — (TIF) [file pone.0191658.s004.tif]

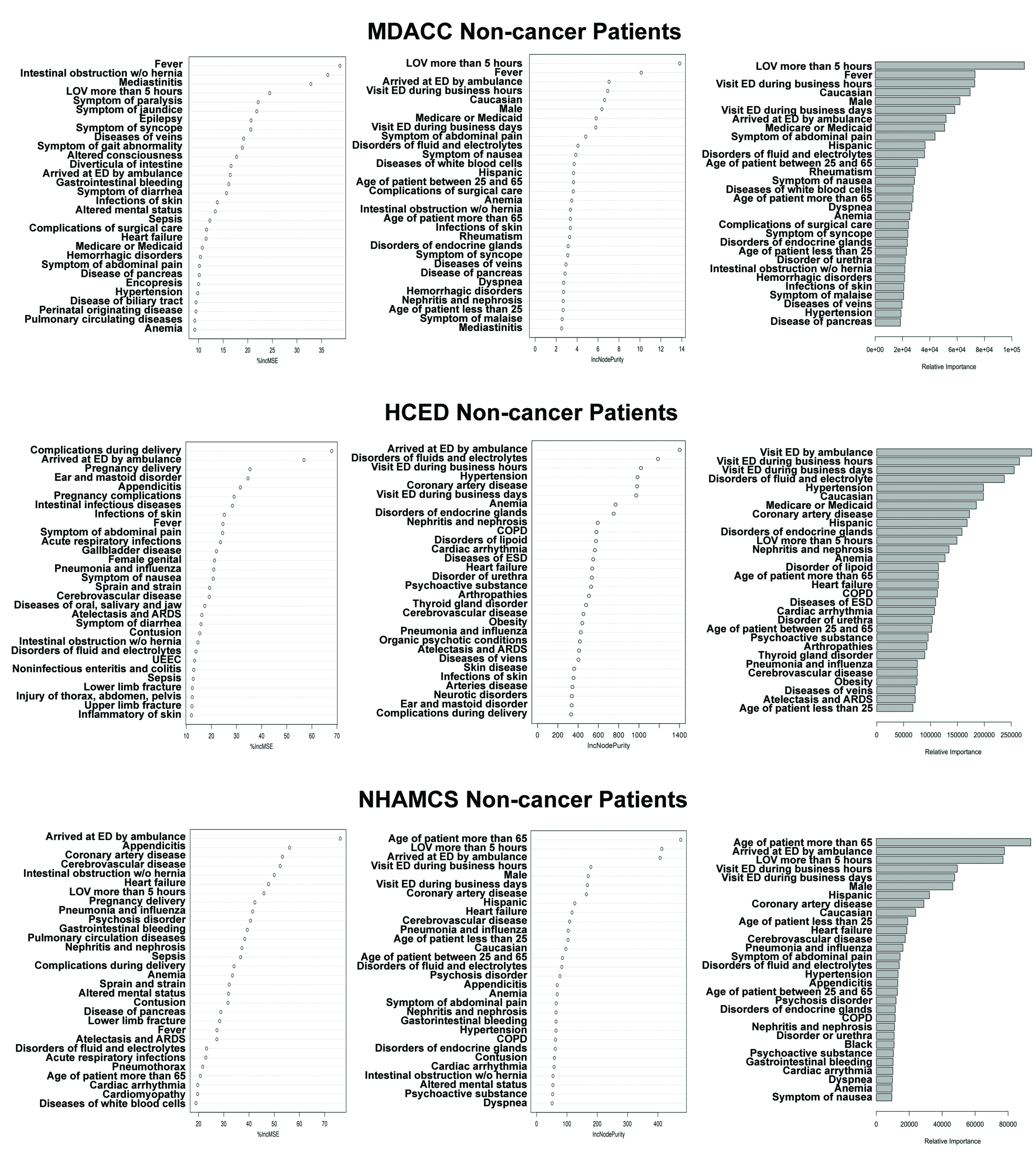

Supplement: S5 Fig — For each database, the top 30 factors associated with the ED admission were ranked by the average percentage increase in mean squared error (%IncMSE) (left panels) or the increase in node purity (IncNodePurity) as calculated by the residual sum of squares (middle panels) using the R package “randomForest”. The relative importance results of factors were identified by random forest using the R package “h2o” (right panels). (TIF) [file pone.0191658.s005.tif]

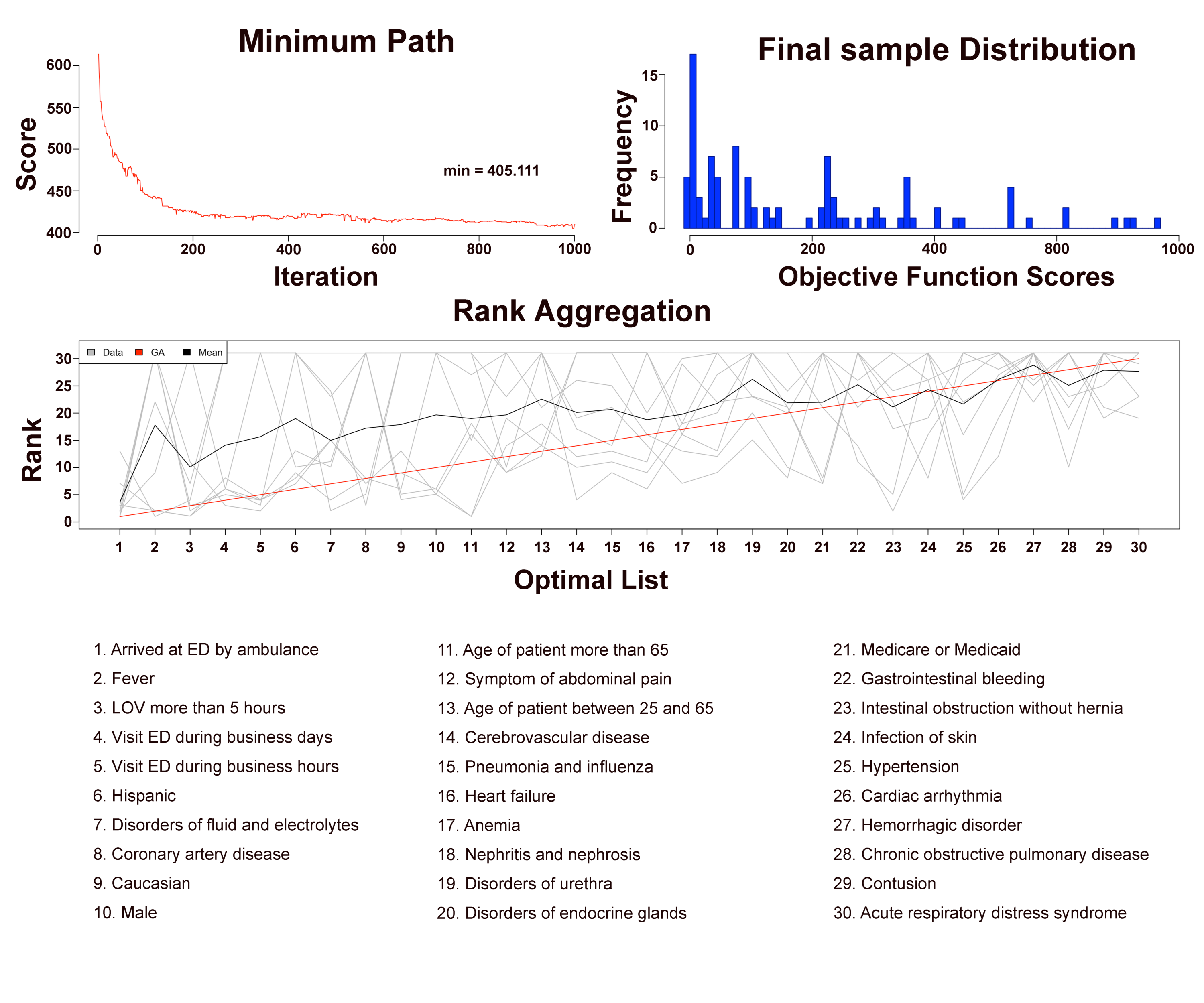

Supplement: S6 Fig — Rank aggregation of the top 30 factors associated with admission through the ED for NCPs who visited the ED (HCED and NHAMCS only). The ranked lists from S5 Fig were aggregated into one list. Result of Rank Aggregation was shown with a genetic algorithm (GA) score of 351.3. (TIF) [file pone.0191658.s006.tif]
